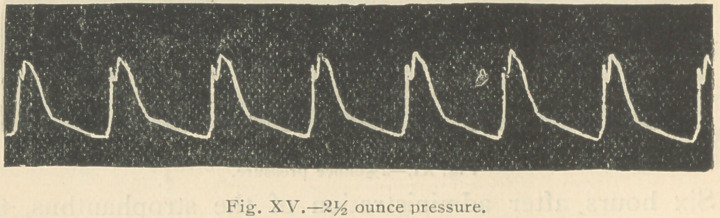# Strophanthus (Hispidus)

**Published:** 1887-03

**Authors:** Charles W. Purdy

**Affiliations:** Professor of Renal Disease at the Chicago Policlinic; 163 State street


THE CHICAGO

### MEDICAL JOURNAL AND EXAMINER.

VOL. LIV. MARCH, 1887. NO. 3

### ORIGINAL COMMUNICATIONS.

STROPHANTHUS (HISPIDUS), ITS PHARMACOLOGY AND THERAPEUTICS. *By* CHARLES W. PURDY, M.D. *Professor of Renal Disease at the Chicago Policlinic.*

Strophanthus is a climbing plant belonging to the natural order of the *Apocynaceæ* and is indiginous to equatorial Africa. The seeds are the most active part of the plant, and when finely ground and made into a paste, are used by several African tribes as an arrow poison. Strophanthine, the active principle of the plant—a crystalline glucoside—is present in the leaves and bark, but to a much less extent than in the seeds.

Attention was first called to the pharmacological action of strophanthus by Pélikan, of St. Petersburg, in a communication to the Academy of Sciences, of Paris, in 1865. During the same year reference was made to it in a note to a paper on certain heart poisons by Fagge and Stevenson of London.

We are chiefly indebted, however, to Prof. Thomas R. Fraser of Edinburgh, for bringing this agent prominently before the profession. At the meeting of the British Medical Association held at Cardiff in June, 1885, Professor Fraser in a most interesting paper*—the result of fifteen years' observations upon man and the lower animals—first clearly pointed out the physiological action of the drug. While ranking strophanthus with the digitalis group as one of the muscle poisons which act, not only upon the heart, but also upon all striped muscular fiber, he also pointed out certain essential differences between the action of strophanthus and that of other members of the group on the circulatory apparatus.

Chief among these differences, and one which is likely to prove of the greatest value therapeutically, is the fact that while strophanthus acts upon the cardiac muscle, even more powerfully as a stimulant than does digitalis, yet the former exerts little or no influence on the arterioles.

It has been conclusively proven by numerous experiments upon frogs, rabbits, etc., that digitalis, while it increases the power of the heart's contractions, also acts as a powerful vaso-constrictor on the arterioles. It is the latter action of digitalis which renders it valuable in the early stages of inflammations. It narrows the channels of the blood in its peripheral distribution, thereby reducing arterial congestion; it also in contracting the vessels (arterioles) restores their lost tone and diminishes the permeability of their coats, thus checking the migration of corpuscles, and the effusion of inflammatory products into the surrounding tissues. But while digitalis acts so happily in controlling many early inflammations, notably those situated in certain structures of a more or less spongy consistence, as the kidneys, lungs etc, yet this same powerful vaso-constrictor action on the vessels increases the labor of the heart by impeding the arterial circulation, and throwing back upon the heart a greater column of blood, the force of which must be met and overcome by the cardiac

*See British Med. Jour. Nov. 14th, 1885

muscle. Now it is readily conceivable how when the cardiac muscle has begun to yield to the weakening influence of organic change—the ultimately legitimate result of every cardiac hypertrophy if only it lasts sufficiently long—and more especially when the aortic valves become incompetent, that the use of digitalis not only hastens the course of the cardiac disease, but it also greatly increases the danger of sudden death. The greater column of blood regurgitating into the left ventricle, backed by a more rigid and unyielding arterial system, more quickly causes the muscular fibres of the ventricle to yield, and thus dilitation is hastened. In addition to this, the increased force of the blood-column resting within and against the ventricle, requires a much slighter re-enforcement (such as may result from a muscular effort) to give it an impetus, the force of which is greater than the ventricle is able to overcome, in which case death becomes the almost instantaneous result.

Valuable as digitalis undoubtedly is in many cardiac affections—especially if the ventricles be strong, and the valves guarding the exit from the heart, the aortics, be intact; yet many careful observers have learned from experience its dangers in the opposite conditions, and have been casting about for some agent which will add strength to the cardiac muscle without lending an additional burden to be borne by the weakened heart. Happily in strophanthus such an agent now seems to be supplied.

In further distinguishing between the physiological action of digitalis and that of strophanthus, Professor Fraser has stated that the latter acts upon the heart the more rapidly as well as the more powerfully of the two. From considerable clinical experience in the use of strophanthus I must add my testimony to that of Professor Fraser's upon this point. From a series of experiments conducted with full doses of tincture of strophanthus—10 minims—I have found pretty uniformly that the sphygmograph indicated a considerable increase of cardiac power in one hour after the administration of the drug; that it reached its maximum in from one and a half to four hours; that the increased power was well maintained from a single dose for eight hours; and that it did not lose its entire effect for twenty-four hours, and often for a longer time. The temperature under the tongue, according to my own observations, begins to fall in about one hour and a half after a 10-minim dose of the strophanthus tincture, and in two hours it usually marks a half to three-fourths of a degree Fahrenheit reduction, maintaining pretty uniformly the above fall for from six to ten or twelve hours, and in some cases longer. I have observed no gastric or intestinal irritation from the use of strophanthus, although I have given it in moderately full doses — 5 to 7 minims of the tincture — for several weeks continuously. Given as above, it has slowed the pulse down as low as 60 beats in the minute. In one case, after its continuous use for a month in pretty full doses, the heart suddenly took on a very rapid action, reaching 150 pulsations per minute. This abnormal rapidity of the heart's pulsations gradually subsided, reaching nearly the normal in about three and a half hours. There was no cardiac pain observed, which is so frequent an accompaniment of the over-action of digitalis.

For comparative purposes, Professor Fraser has conducted experiments upon the frog's heart, by supplying it with solutions both of digitaline and strophanthine by means of Williams' apparatus. He found that a solution of digitaline,

1 part in 4,000, was not sufficient to kill the heart; while on the other hand, the enormous dilution of strophanthine of 1 part in 10,000,000 was strong enough to kill (arrest) the heart. A solution of one part strophanthine in 6,000,000 produced complete stoppage of the heart's contractions in extreme systole in twenty minutes.

With regard to the action of strophanthus on the vessels (arterioles) Professor Fraser employed solutions of digitaline and strophanthine upon the frog after destruction of the central nervous system. In the case of digitaline, 1 part in 20,000, produced in six or seven minutes such extreme contraction of the vessels as to arrest the circulation through them.

Substituting strophanthine for digitaline, 1 part in 20,000, had no effect upon the vessels, and when the strength was increased to 1 part in 2,000 but a temporary effect was produced, which soon subsided.

A patient of mine, suffering from advanced mitral and aortic disease, was given a single dose of 10 minims of the tincture of strophanthus, the patient remaining quietly in bed for some time before, and during the whole time of observations no medicines nor stimulants were given save the strophanthus. Before the strophanthus was administered the pulse was 78 per minute, the respirations were 18 per minute, and the temperature under the tongue was 98.5° F. The following tracings were taken (Figs. 1 and 2):

In Fig. 1 just sufficient pressure was applied—½ ounce—to give the greatest freedom of movement to the lever. It will be observed that when the pressure upon the artery was increased to 2 ounces, the tracing became almost reduced to the respiratory or base line, so weak was the muscular power of the ventricle.

Fig I.—½ ounce pressure.

Fig. II.—2 ounce pressure.

One hour after the 10 minims of strophanthus tincture was given the pulse was 75 per minute, the respirations were 16 per minute, and the temperature under the tongue was 98.5° F. The tracings 3, 4, and 5 were then taken.

Fig. III.—1½ ounce pressure.

Fig. IV.—2 ounce pressure.

It will be observed (Fig. 4) that two ounces pressure upon the artery scarcely modified the tracing, that even 2½

ounces pressure (Fig. 5) failed to materially lower the apex of the tracing, so markedly increased had become the power of the ventricular contraction.

Fig. V.—2½ ounce pressure.

Two hours after the 10 minims of strophanthus tincture was given, the pulse was 72 per minute, the respirations were 18 per minute, and the temperature under the tongue was 97.8° F.; the temperature had therefore fallen .7° F. in two hours. The following tracings were then taken:

Fig. VI.—2 ounce pressure.

Fig. VII.—3 ounce pressure.

It will be observed (Fig. 6) that under 2 ounces pressure (which before the strophanthus was given nearly reduced the tracing to the respiratory line), the freedom of the lever was now scarcely modified, and that even 3 ounces pressure upon the artery (Fig. 7) but little modified the height of the tracing.

Three hours after the 10-minim dose of strophanthus was administered, the pulse was 72 per minute, the respirations were 17 per minute, and the temperature under the tongue was 97.8° F. The following tracings were then taken Figs. 8 and 9):

Fig. VIII.—2 ounce pressure.

Fig. IX.—3½ ounce pressure.

Four hours after the strophanthus was given, the pulse was 69 per minute, the respirations were 18 per minute, and

Fig. X.—1½ ounce pressure.

the temperature under the tongue was 97.8° F. The accompanying tracings were then taken (Figs. 10 and 11).

Fig. XI.—2½ ounce pressure.

Six hours after administration of the strophanthus, the pulse was 69 per minute, the respirations were 18 per minute, and the temperature under the tongue was 97.8° F. The following tracings (Figs. 12 and 13) were then taken:

Fig. XII.—2 ounce pressure.

Fig. XIII.—2½ ounce pressure,

Eight hours after administration of the strophanthus, the pulse was 72 per minute, the respirations were 16 per

Fig. XIV.—1½ ounce pressure.

minute, and the temperature under the tongue was 97.6° F.

The accompanying tracings (Figs. 14 and 15) were then taken:

Fig. XV.—2½ ounce pressure.

It will be observed that at the end of eight hours after administration of a single dose of 10 minims of the tincture of strophanthus, the drug maintained a decided action upon the heart and temperature.

Although I have a large number of sphygmographic tracings of the pulse after the use of strophanthus, I have selected the foregoing case as probably the best one to illustrate the action of strophanthus upon the ventricular muscle for the following reasons: The case was one of grave aortic defect, and therefore the pressure upon the artery showed more plainly the increase of cardiac power induced by the drug than if the aortic valves were intact, for obvious reasons. It will be noted of the sphygmograms taken after the fourth hour from the administration of strophanthus that the tidal wave became very marked, showing a tendency to rigidity in the ventricular muscle in systole; the more gradual slope of the tracing following the aortic wave points to the same condition of the heart in diastole.

The action of strophanthus as a diuretic is scarcely less pronounced than as a cardiac stimulant; in fact the former depends upon the latter. I hope in a future communication to dwell more fully upon this part of the subject.

To sum up briefly then, strophanthus seems to act upon the heart more powerfully and more rapidly than does digitalis. "In lethel doses it paralyzes the heart in rigid systole." It acts but slightly upon the vessels, and only in large doses. It lowers the normal temperature from .5° F. to .1° F. It causes a direct and marked rise in the arterial blood pressure, and thus acts as a diuretic.

With the foregoing data to guide us, the very practical question next presents itself, What are the therapeutic indications for the use of strophanthus?

There can be no question but that we have much yet to learn upon this point, and for what we already know we are largely indebted to Professor Fraser, as we undoubtedly are for our knowledge of the pharmacology of the drug.

Nearly a year ago I strongly advised* the use of strophanthus in the late stages of heart affections which so commonly accompany chronic Bright's disease (pp. 187), also in cardiac collapse common to scarlatinal nephritis (pp. 223), and lastly in so-called cyanotic induration of the kidney (pp. 279).

In the last named condition especially, where the venous system is over-distended at the expense of the arterial, I have found it the most useful of all agents. Of heart disease in general it may be said of strophanthus that if not always indicated, it is yet rarely if ever contra-indicated. In all weakened conditions of the heart then, if the cardiac muscle has not undergone organic change, strophanthus promises much. If the cardiac muscle has undergone fatty change, of course no agent is capable of restoring its power; but as the exact state of the cardiac muscle with regard to organic change can never be accurately, determined, save at the autopsy, we may

* Bright's Disease and Allied Affections of the Kidney. Philadelphia: Lea Bros. & Co. London: H. K. Lewis.

yet give strophanthus when the condition is suspected, and not without hope. It at least in all probability has the virtue of working no mischief.

Sir Andrew Clark has reported in a recent article in the *British Medical Journal* over 600 cases of valvular diseases of the heart, selected from his private practice, none of which lasted less than five and many of them over twenty-five years. It is significant that in a large number of the cases the patients went about their daily duties without regular treatment so far as drugs are concerned. This strongly suggests to me the necessity of at least using agents in cardiac affections which are not likely to work harm.

Mitral disease of the heart—either stenosis or insufficiency—is likely to furnish a most promising field for the therapeutic use of strophanthus. Given early it is likely to retard hypertrophy more efficiently than digitalis. Under the use of strophanthus the cardiac muscle is much less prone to pass into fatty change than under the use of digitalis, because in the former case the vessels remaining uncontracted permit a better supply of blood to, and consequently a better nourishment of, the cardiac muscle.

In threatened collapse of the heart's action from most causes strophanthus is strongly indicated; and in such cases it should be given sub-cutaneously in the form of strophanthine.

Strophanthus is perhaps the most promising agent we possess for the relief of cardiac dropsy with its usually attendant dyspnœa, both of which it often relieves with promptness and efficiency.

Strophanthus should not be used in acute inflammation, possibly excepting that of the lungs. Especially should its use be avoided in the treatment of acute nephrites accompanied by bloody urine.

Dr. Smith of Dublin,* while he considers strophanthus a valuable drug for controlling the heart's action, yet he does not consider it suitable for the use of every patient. I have noted in some cases that it seems to irritate the heart, though usually not unless full doses are administered.

For the benefit of those who are unfamiliar with strophanthus I shall in conclusion briefly consider the pharmacy of the drug. This indeed seems the more necessary since heretofore different strengths of the tincture have been put upon the market.

Professor Fraser originally advised the use of a tincture of the same strength as that of digitalis, i. e. 1 in 8. He now, however, advises for general use a tincture of the strength of 1 to 20. In my recent work on renal diseases I uniformly advised a dose of from 2 to 6 drops, basing my calculations upon Professor Fraser's original formula. According to his new formula the above dose should be increased two and a half times—5 to 15 drops. Professor Fraser now gives the following directions for preparing strophanthus tincture.†

" Strophanthus seeds, deprived of their comose appendices, reduced to powder and dried, 1 ounce or 1 part. Ether, freed from spirit and from water, 10 fluid ounces, or fluid parts. Rectified spirit, a sufficiency to obtain 1 pint, or 20 fluid parts.

"Remove entirely the stalks and comose appendices from the seeds, reduce the seeds to a moderately fine powder, dry the powder by exposing for twelve hours to a temperature of 100° or 120° F., and weigh. Pack in a percolator (the percolator being furnished with air valves, or being otherwise so constructed that the percolation may be arrested

* Year Book of Treatment for 1886, pp. 293.
† See British Medical Journal, Jan. 22d, 1887.

when desired), add ether until the whole of the powder is saturated, and a small quantity of the ether has dropped into the percolator; arrest the percolation for 24 hours, and then continue percolating slowly until the whole of the ether has been used. If the last ether percolate should not be almost colorless, use more ether."

"Remove the powder from the percolator; expose to the air, and break up any lumps after the ether has sufficiently evaporated; and continue the exposure, heating the powder, if necessary, to 100° or 120° F., until all the ether has evaporated, when a uniform, nearly white, dry powder may easily be obtained."

" Repack the powder in the percolator, add enough rectified spirit to moisten it thoroughly; arrest the further flow of the spirit, and macerate for 48 hours; and pass rectified spirit slowly through until twenty fluid pints of tincture are obtained.

"In this process, the preliminary extraction with ether is for the purpose of removing the large quantity of inert oil contained in the seeds, which, if present in the tincture, would cause it to become opalescent on the addition of water."

The above tincture is nearly colorless, having a very pale yellow tinge (not green), neutral in reaction and intensely and rather persistently bitter to the taste. It mixes unchanged with water, is not precipitated by tannin, but becomes opalescent upon the addition of ether. Solution of ferric perchloride intensifies its yellow color, and produces a slight haziness in the solution, and after some hours it turns a greenish yellow color. The dose of the above tincture is from 2 to 10 minims (5 to 30 drops). If tincture of strophanthus be slowly propelled from an ordinary nipple medicine dropper it will drop from 2 to 3 drops to the minim, according to the size of the tube at its point.

In general the dose which has yielded me the best result. has been 4 to 6 minims, repeated about three times in 24 hours (every 8 hours). It is a useful method occasionally to suspend the use of the drug for 24 or 48 hours in order to more accurately determine its effects upon the heart. I have given 10-minim doses of strophanthus tincture (1 in 20) every 6 hours for two or three days without serious disturbances The heart was, however, stimulated to rather violent though regular action; the ordinary paper for sphygmographic use (1 inch in width) not being sufficiently wide to take the whole stroke of the tracing lever.

Messrs. E. H. Sargent & Co., 125 State street, have kindly prepared for me a tincture of strophanthus according to the before described formula of Professor Fraser, and it is now kept in their stock. Parke, Davis & Co., of Detroit, have also furnished me with a tincture of strophanthus which has given satisfactory results.

163 State street.

## Figures and Tables

**Fig I. f1:**
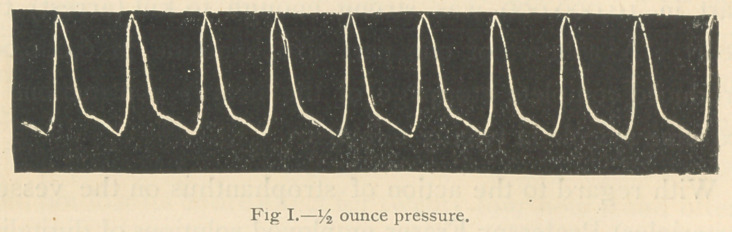


**Fig. II. f2:**
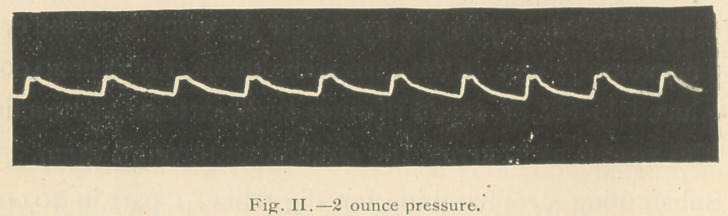


**Fig. III. f3:**
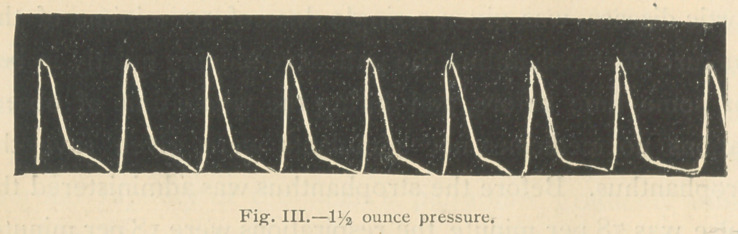


**Fig. IV. f4:**
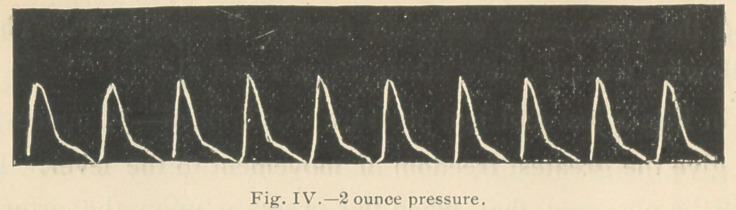


**Fig. V. f5:**
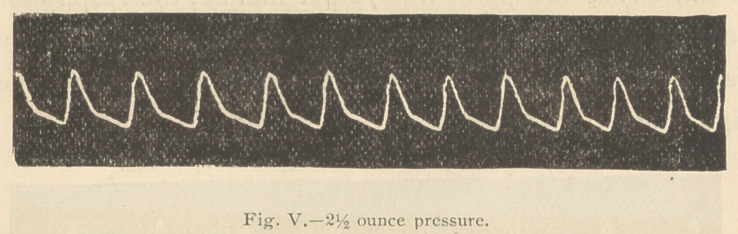


**Fig. VI. f6:**
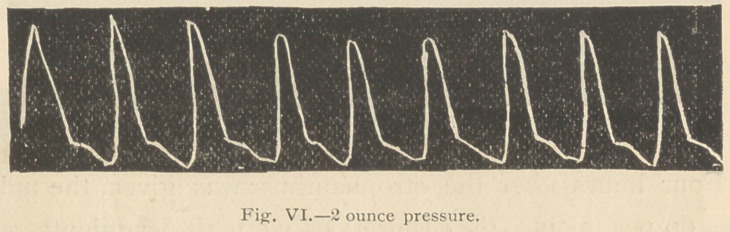


**Fig. VII. f7:**
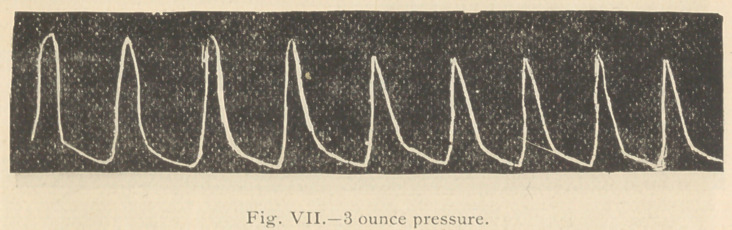


**Fig. VIII. f8:**
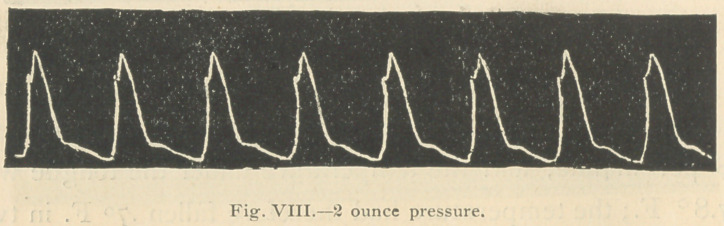


**Fig. IX. f9:**
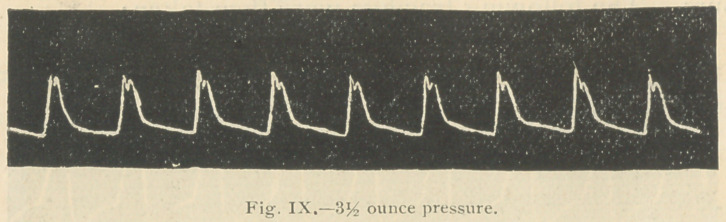


**Fig. X. f10:**
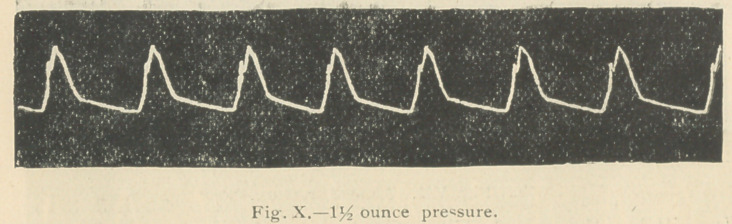


**Fig. XI. f11:**
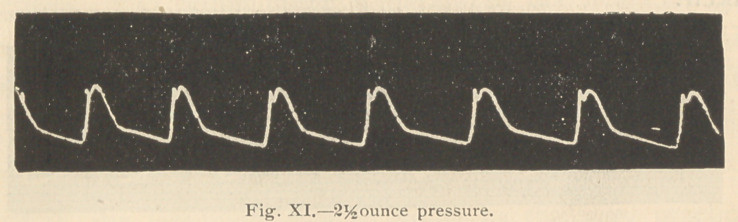


**Fig. XII. f12:**
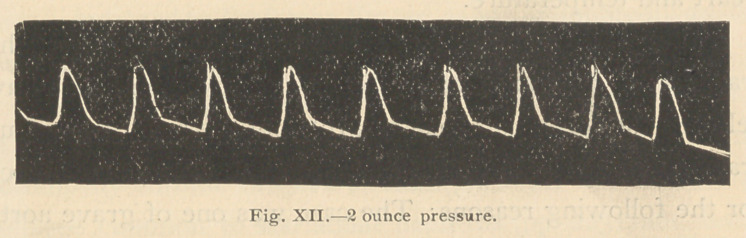


**Fig. XIII. f13:**
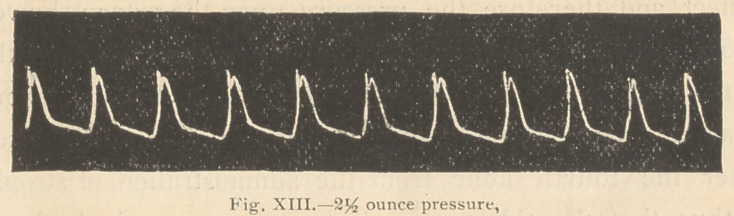


**Fig. XIV. f14:**
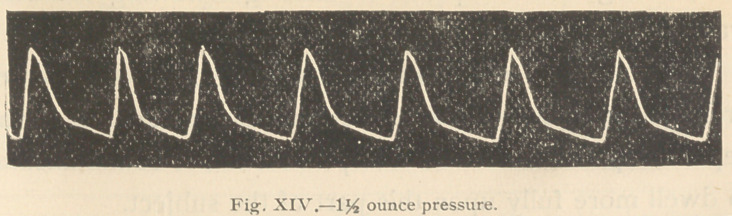


**Fig. XV. f15:**